# Resistance pattern of isolated microorganisms from 783 clinical specimen cultures in patients admitted to Yasuj Educational Hospitals, Iran

**DOI:** 10.1186/s12866-023-02952-4

**Published:** 2023-08-01

**Authors:** Fatemeh Forouzani, Tahere Khasti, Leila Manzouri, Sara Ravangard, Reza Shahriarirad, Maryam Koleini, Nazanin Ayareh, Gordafarin Nikbakht

**Affiliations:** 1 School of Medicine, Yasuj University of Medical Sciences, Yasuj, Iran; 2 Social Determinants of Health Research Center, Yasuj University of Medical Sciences, Yasuj, Iran; 3 Thoracic and Vascular Surgery Research Center, Shiraz University of Medical Science, Shiraz, Iran; 4 Department of Microbiology, Faculty of Medicine, Shahid Sadoughi University of Medical Sciences, Yazd, Iran; 5 Students Research Committee, School of Medicine, Shiraz University of Medical Sciences, Shiraz, Iran

**Keywords:** Antibiotic, Antimicrobial, Antimicrobial resistance, AMR, Bacteria, Drug Resistance, Iran

## Abstract

**Background:**

Infectious diseases are still one of the leading causes of morbidity and mortality in resource-limited settings. Serious infection caused mostly by gram-negative pathogens causes significant morbidity. According to the Centers for Disease Control and Prevention, antimicrobial resistance kills over 700,000 people worldwide. Antibiotic resistance is on the rise, and as a consequence, serious public health issues are arising. The present study investigated isolated clinical samples from Yasuj teaching hospitals to determine the antimicrobial resistance profile to various antibiotics.

**Materials and methods:**

Microbial isolates regarding cultures from urine, blood, wound, abdominal tap, throat, stool, cerebrospinal fluid, endotracheal tube, sputum, skin lesion, nasal, and mouth secretion were collected from patients admitted to hospitals affiliated with Yasuj teaching hospitals. Antibiotic susceptibility profiles were determined by using the Kirby-Bauer disc diffusion method. Data were tabulated and analyzed with SPSS version 26.0.

**Results:**

A total of 783 samples were evaluated in our study, with an average of 30.6 years and 54.5% female patients. Most of the bacterial isolates were gram-negative (64.2%). The majority of cultures were *Escherichia coli* (49.9%), mainly among urine samples (64.2%). The frequency distribution of norfloxacin antibiotic resistance was more common in internal medicine (66.7%), infectious (63.6%), and emergency wards (58.8%). The frequency distribution of penicillin antibiotic resistance was statistically significant in different wards. All cases of oxacillin were resistant.

**Conclusion:**

Our data showed a high level of antibiotic resistance among bacterial isolates in our center. Considering widespread empirical antibiotic therapy in Iran, the rate of increasing resistance to common antibiotics prescribed for ambulatory and hospitalized patients is concerning. We recommend providing more strict guidelines and policies to control the overuse and overprescription of antimicrobials by health policy-making organizations.

## Introduction

Infectious diseases are still one of the leading causes of morbidity and mortality in resource-limited settings, but epidemiological data on causative pathogens and antimicrobial resistance (AMR) distribution are scarce [1]. Serious infection caused mostly by gram-negative pathogens causes significant morbidity, and emerging multidrug resistance patterns increase morbidity and mortality even further, especially in critically ill patients [2–4]. According to the Centers for Disease Control and Prevention, antimicrobial resistance kills over 700,000 people worldwide each year, with that figure expected to rise to 10 million by 2050 [5]. Physicians are increasingly challenged to provide their patients with effective antibiotic regimens that do not result in further drug resistance. Antibiotic resistance is on the rise, and as a consequence, serious public health issues are arising [6, 7]. New antimicrobial pharmaceutical investigations are desperately needed and are a primary goal. Updating knowledge on common antimicrobial-resistant pathogens’ occurrence and resistance may help establish better antibiotic use in outpatient and inpatient settings. In the present study, clinical samples isolated from Yasuj teaching hospitals were investigated to determine the antimicrobial resistance profile to various antibiotics.

## Method

In this multicentric, cross-sectional, descriptive-analytical study, bacterial isolates were identified using phenotypic methods including: observation of growth and colony morphology on various media and analysis of biochemical reactions [8]. All positive cultures of clinical samples reported in teaching hospitals affiliated with Yasuj University of Medical Science during a one-year period (2017–2018) were included. After obtaining the approval of the ethics committee, required information regarding positive blood culture samples were drawn from the referrals to the laboratories of Imam Sajjad and Shahid Beheshti hospitals. All isolates’ antibiotic susceptibility profiles were determined by investigating antibiotic disks using Kirby–Bauer disc diffusion method using Muller-Hinton Agar (Merck) as a solid medium [9]. Clear zones (zones of inhibition) were measured after 18 h by a scale and then compared to a Clinical and Laboratory Standard Institute (CLSI) chart, which contains information of standard measurements that indicate the particular sample is sensitive, intermediate or resistant to a specific antibiotic [10]. All positive cultures were included in this study except for the incomplete registration information or the growth of various microorganisms in favor of contamination. A contaminated urine culture is defined as the presence of more than 2 isolates at greater than or equal to 10,000 CFU/mL and must be excluded from the study.

Specimens were collected from urine, blood, wound, abdominal tap, throat, stool, cerebrospinal fluid (CSF), endotracheal tube (ETT), sputum, skin lesion, nasal, and mouth secretion. At least 60 ccs of midstream urine and 30 cc of blood were collected from each adult participant for culture. Other samples including wounds were collected using appropriate sterile sampling equipment (swabs, sterile syringes, etc.)

The tested antibiotics included: Norfloxacin(10 µg), Nitrofurantoin(300 µg), Penicillin(10units), Ciprofloxacin(5 µg), Cotrimoxazole(1.25/23.75 µg), Ceftazidime(30 µg), Ofloxacin(5 µg), Cefixime(5 µg), Cephalothin(30 µg), Amikacin(30 µg), Ceftriaxone(30 µg), Gentamycin(10 µg), Cefalexin(30 µg), Imipenem(10 µg), Cefotaxime(30 µg), Cefazolin(30 µg), Ampicillin(10 µg), Co-Amoxiclav(20/10µg), Rifampin(5 µg), Tetracycline(30 µg), Azithromycin(15 µg), Vancomycin(30 µg), Erythromycin(15 µg), Clindamycin(2 µg), Nalidixic Acid(30 µg), Ceftizoxime(30 µg), Cefoxitin(30 µg), Amoxicillin(30 µg), Cefepime(30 µg), Doxycycline(30 µg), and Oxacillin(1 µg). Antimicrobial susceptibility tests were based on the CLSI guidelines [10].

The collected data were analyzed using SPSS software version 26 using descriptive statistics (mean, standard deviation (SD), or frequency and percentage (%)) and analytical tests. Categorical variables were evaluated with Chi-square and Fisher’s exact test. A P-value of less than 0.05 was considered statistically significant.

## Results

A total of 783 samples were evaluated in our study, with their age ranging from 1 day to 97 years old, with an average of 30.6 years and 54.5% females. Most of the bacterial isolates were gram-negative (64.2%). Table 1 demonstrates the baseline features of the patients in our study.

**Table 1:** Baseline hospital features of patients in our study

Variable		Frequency (%)
Age group	≤ 6	333 (42.5)
	7–18	35 (4.5)
	18–60	216 (27.6)
	> 60	199 (25.4)
Gender	Male	356 (45.5)
	Female	427 (54.5)
Hospital	Shahid Beheshti	247 (31.5)
	Imam Sajjad	536 (68.5)
Bacteria type	Gram-negative	503 (64.2)
	Gram-positive	280 (35.8)
Specimen	Wound	44 (5.6)
	Urine	519 (66.3)
	Blood	143 (18.3)
	Tap	16 (2.0)
	Throat	12 (1.5)
	Stool	9 (1.1)
	CSF	3 (0.4)
	ETT	6 (0.8)
	Sputum	8 (1.0)
	Skin Lesion	2 (0.3)
	Nasal	6 (0.8)
	Mouth secretion	7 (0.9)
	Other	8 (1.0)
Ward	Intensive Care Unit	32 (4.1)
	Surgery	41 (5.2)
	Internal	148 (18.9)
	Infectious	66 (8.4)
	Burn	12 (1.5)
	Emergency	80 (10.2)
	Neonatal	72 (9.2)
	Coronary Care Unit	3 (0.4)
	Postpartum/Labor	58 (7.4)
	Pediatric Emergency	271 (34.6)

The patient’s specimen and culture results were evaluated and the results of AMR are demonstrated in Table 2.

**Table 2:** Antibiotic resistance patterns and their association with age, gender, and admission ward based on antibiotic pattern evaluation

Antibiotic	Total	Pattern*			P-value			
		Sensitive	Intermediate	Resistant	Age	Gender	Ward	
Amikacin	328 (41.9)	236 (72.0)	42 (12.8)	50 (15.2)	**0.028**	**0.004**	**< 0.001**	
Amoxicillin	212 (27.1)	32 (15.1)	14 (6.6)	166 (78.3)	**0.005**	0.805	**0.035**	
Ampicillin	119 (15.2)	27 (22.7)	21 (17.6)	71 (59.7)	0.201	0.563	0.228	
Azithromycin	23 (2.9)	1 (4.3)	1 (4.3)	21 (91.3)	0.349	0.167	0.329	
Cefalexin	185 (23.6)	57 (30.8)	15 (8.1)	113 (61.1)	**0.009**	0.368	**< 0.001**	
Cefazoline	105 (13.4)	29 (27.6)	12 (11.4)	64 (61.0)	0.851	0.643	**0.004**	
Cefepime	37 (4.7)	20 (54.1)	5 (13.5)	12 (32.4)	0.127	0.701	0.809	
Cefixime	272 (34.7)	89 (32.7)	13 (4.8)	170 (62.5)	**0.019**	0.494	**0.006**	
Cefotaxime	179 (22.9)	67 (37.4)	19 (10.6)	93 (52.0)	**0.021**	0.645	**< 0.001**	
Cefoxitin	64 (8.2)	26 (40.6)	3 (4.7)	35 (54.7)	0.055	**0.036**	0.311	
Ceftazidime	51 (6.5)	17 (33.3)	6 (11.8)	28 (54.9)	0.523	0.205	0.379	
Ceftizoxime	27 (3.4)	11 (40.7)	2 (7.4)	14 (51.9)	0.728	1.000	0.587	
Ceftriaxone	278 (35.5)	108 (38.8)	20 (7.2)	150 (54.0)	**0.045**	**0.030**	0.055	
Cephalothin	99 (12.6)	31 (31.3)	7 (7.1)	61 (61.6)	0.780	0.277	0.708	
Ciprofloxacin	344 (43.9)	177 (51.5)	29 (8.4)	138 (40.1)	**0.003**	0.156	**0.012**	
Clindamycin	67 (8.6)	22 (32.8)	10 (14.9)	35 (52.2)	0.120	0.293	0.134	
Co-Amoxiclav	13 (1.7)	5 (38.5)	1 (7.7)	7 (53.8)	0.437	0.624	0.125	
Cotrimoxazole	386 (49.3)	115 (29.8)	8 (2.1)	263 (68.1)	0.061	0.721	0.930	
Doxycycline	9 (1.1)	7 (77.8)	0 (0)	2 (22.2)	0.764	0.167	0.049	
Erythromycin	63 (8.0)	14 (22.2)	7 (11.1)	42 (66.7)	0.651	0.255	0.512	
Gentamycin	632 (80.7)	424 (67.1)	57 (9.0)	151 (23.9)	0.065	**0.014**	**< 0.001**	
Imipenem	46 (5.97)	24 (53.3)	10 (22.2)	11 (24.4)	**0.018**	0.903	0.318	
Nalidixic Acid	183 (23.4)	41 (22.4)	29 (15.8)	113 (61.7)	0.685	0.197	0.732	
Nitrofurantoin	361 (46.1)	246 (68.1)	58 (16.1)	57 (15.8)	0.069	0.063	**0.003**	
Norfloxacin	140 (17.9)	71 (50.7)	5 (3.6)	64 (45.7)	**< 0.001**	**0.004**	**0.003**	
Ofloxacin	134 (17.1)	56 (41.8)	4 (3.0)	74 (55.2)	**0.032**	**0.033**	0.544	
Oxacillin	5 (0.6)	0 (0)	0 (0)	5 (100)	-	-	-	
Penicillin	189 (24.1)	51 (27.0)	18 (9.5)	120 (63.5)	0.626	0.194	**0.017**	
Rifampin	20 (2.6)	17 (86.0)	0 (0)	3 (15.0)	**0.039**	0.270	0.499	
Tetracycline	65 (8.3)	25 (38.5)	8 (12.3)	32 (49.2)	0.531	0.718	0.151	
Vancomycin	614 (78.4)	52 (30.8)	42 (24.9)	75 (44.4)	0.179	0.740	0.060	

* For pattern, the clear zones (zones of inhibition) were measured after 18 h by a scale and then compared to a CLSI chart, which contains information of standard measurements that indicate the particular sample is sensitive, intermediate or resistant to a specific antibiotic.

As demonstrated in Table 2, the frequency distribution of norfloxacin antibiotic resistance was statistically significant in different wards and was more common in internal medicine (66.7%) and infectious (63.6%), wards. (P = 0.003) The resistance was also significantly higher among the above 60 years age group (P < 0.001).

The frequency distribution of Penicillin antibiotic resistance was statistically significant in different wards (P < 0.05). The highest resistance was related to the maternity ward, infectious ward, and neonates (P = 0.017). Regarding Cefixime, the majority of resistant were among the surgery (79.2%) and infectious (73.3%) wards (P = 0.006). Regarding Amikacin, the majority of sensitive were in the emergency (71.4%), pediatric emergency (84.9%) wards (P < 0.001). The majority of sensitivity regarding Gentamycin was among the postpartum (80.4%) and pediatric emergency (78.5) wards (P < 0.001). Cephalexin resistance majority were in infectious (83.3%), surgery (81.8%), and Intensive care unit (ICU) (75%) wards (P < 0.001). Cefotaxime and Cefazolin were 100% resistant in the ICU (100%) ward (P < 0.001 and 0.004, respectively) and Amoxicillin resistance was mainly in the ICU (80%) (P = 0.035). All cases of Oxacillin were resistant.

**Fig. 1:**
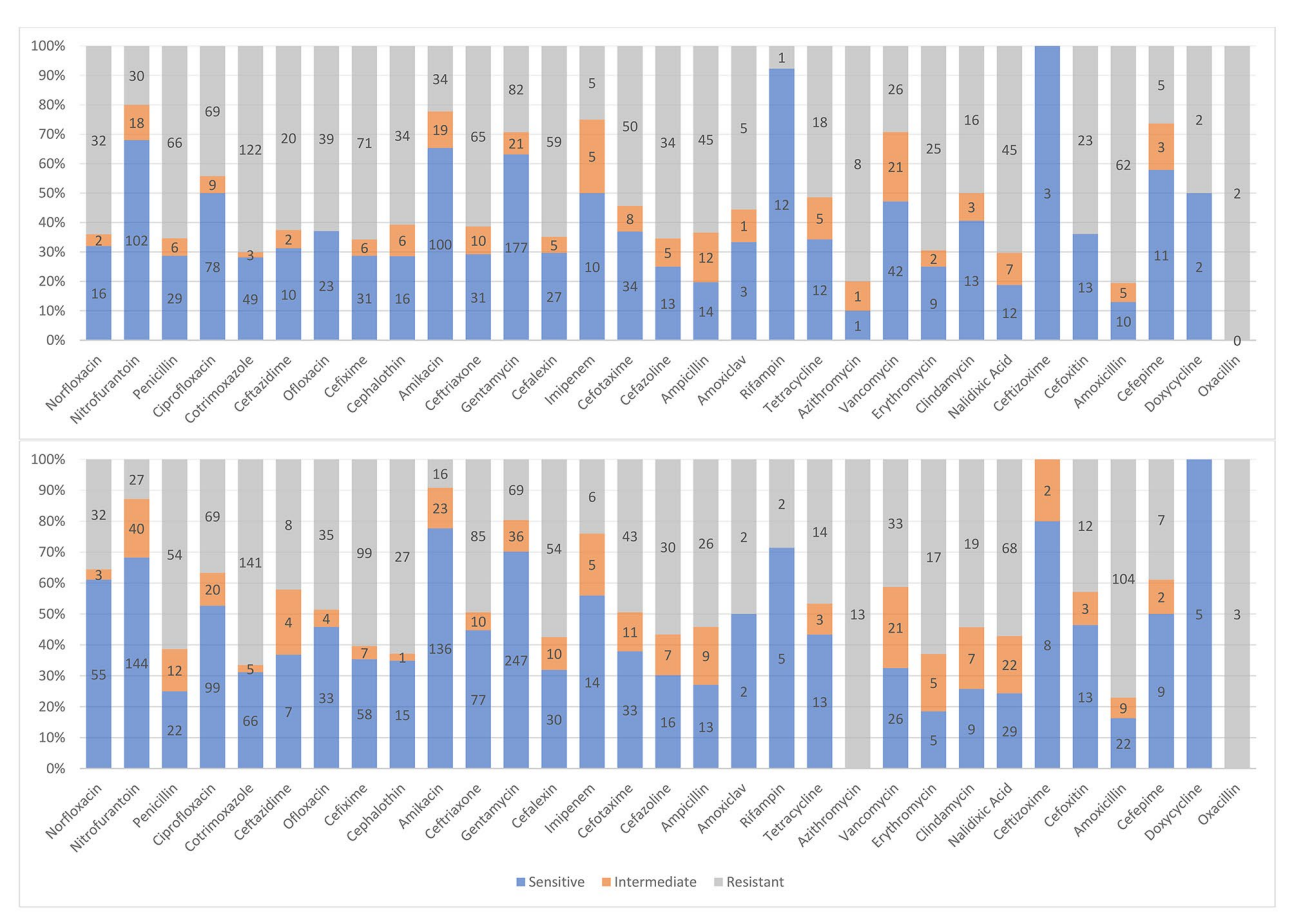
Demonstration of microbiological resistance pattern among male (above) and female (below) patients

**Fig. 2:**
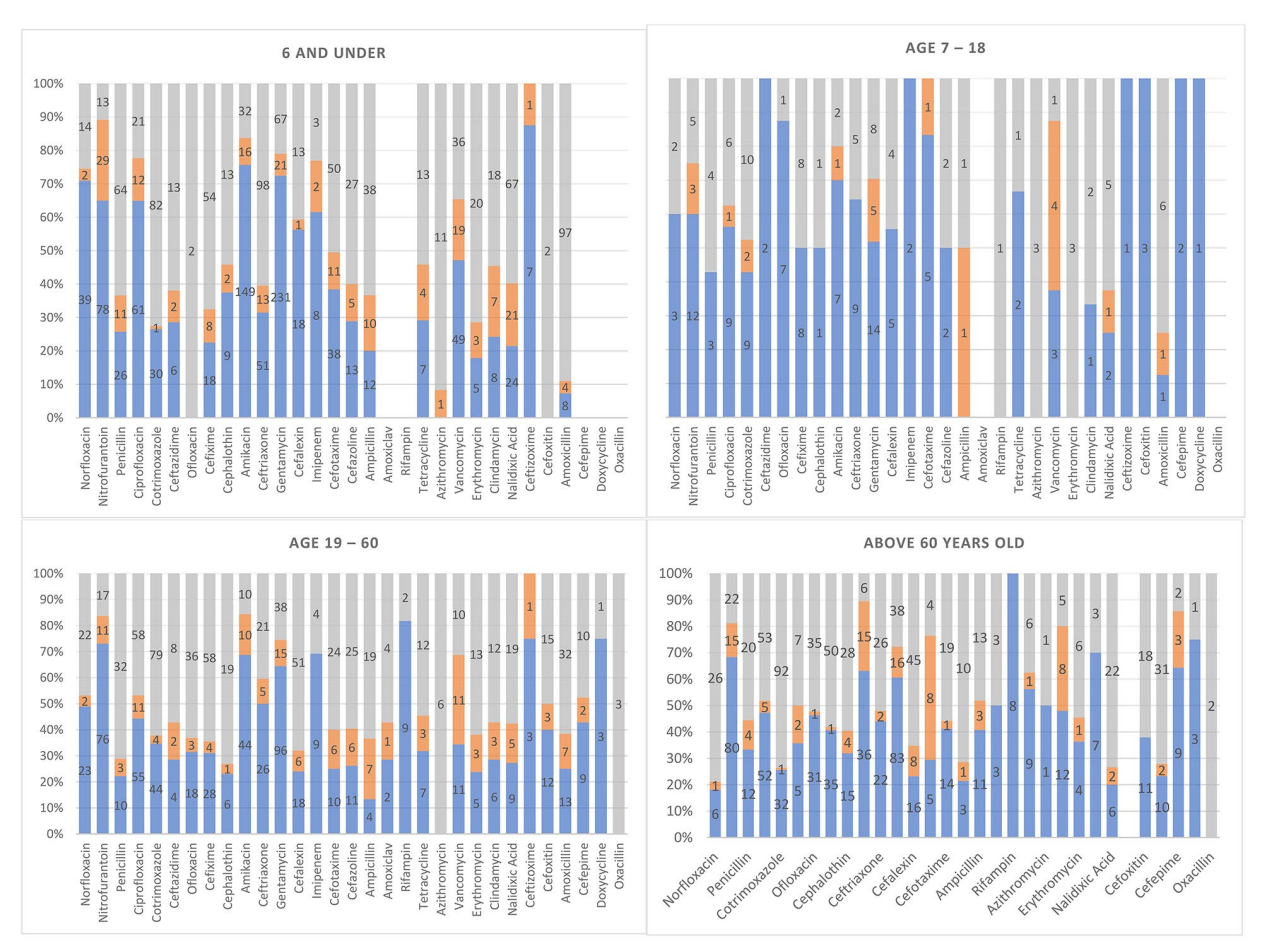
Demonstration of microbiological resistance pattern based on age groups

Figures 1 and 2 demonstrates the distribution of AMR based on the age groups and gender of our patients.

The microbial results of the cultures are presented in Table 3. As demonstrated, most cultures were *Escherichia coli (E. coli)* (49.9%), mainly among urine samples (64.2%). Also, the microbial antibiogram resistance pattern is demonstrated in Tables 4 and 5.

**Table 3:** Evaluation of microbial results based on obtained cultures

Microorganism; n (%)		Wound; n = 44	Urine; n = 519	Blood; n = 143	Tap; n = 6	Throat; n = 12	Stool; n = 9	Cerebrospinal fluid; n = 3	Endotracheal tube; n = 6	Sputum; n = 8	Skin Lesion; n = 2	Nasal; n = 6	Mouth secretion; n = 7	Other; n = 8
*E. coli*	391 (49.9)	14 (31.8)	333 (64.2)	18 (12.6)	4 (25.0)	5 (41.7)	7 (77.8)		1 (50.0)		1 (50.0)	4 (66.7)	1 (14.3)	3 (37.5)
*Staph coagulase negative*	175 (22.3)	12(27.3)	44 (8.5)	11 (12.6)	2 (12.5)	2 (16.7)		1 (33.3)		5 (62.5)				
*Staph coagulase positive*	77 (9.8)	12 (27.3)	58 (11.2)	89 (62.2)	3 (18.8)	2 (16.7)		2 (66.7)		1 (12.5)	1 (50.0)		2 (28.6)	5 (62.5)
*Klebsiella*	74 (9.5)	3 (6.8)	49 (9.4)	6 (4.2)	4 (25.0)	1 (8.3)	1 (11.1)		5 (83.3)			2 (33.3)	3 (42.9)	
*Streptococcus*	28 (3.6)		11 (2.1)	13 (9.1)		2 (16.7)								
*Enterobacter*	14 (1.8)		11 (2.1)		1 (6.3)		1 (11.1)						1 (14.3)	
*Pseudomonas aeruginosa*	12 (1.5)	1 (2.3)	8 (1.5)	2 (1.4)	1 (6.3)					2 (25.0)				
*Proteus*	5 (0.6)	1 (2.3)	4 (0.8)											
*Citrobacter*	4 (0.5)			3 (2.1)	1 (6.3)									
*Alkalogenic bacteria*	1 (0.1)	1 (2.3)												
*Flavobacteria*	1 (0.1)			1 (0.7)										

**Table 4:** Antibiotic resistance patterns based on pathogens

Antibiotic; n (%)	*E. coli*			*Staph coagulase positive*			*Staph coagulase negative*			*Proteus*			*Pseudomonas aeruginosa*			*Klebsiella*		
	Sensitive	Intermediate	Resistant	Sensitive	Intermediate	Resistant	Sensitive	Intermediate	Resistant	Sensitive	Intermediate	Resistant	Sensitive	Intermediate	Resistant	Sensitive	Intermediate	Resistant
Norfloxacin	45 (58.4)	3 (3.9)	29 (37.3)	9 (25.7)	1 (2.9)	25 (71.4)	4 (36.4)		7 (63.6)							7 (87.5)		1 (12.5)
Nitrofurantoin	194 (74.9)	43 (16.6)	22 (8.5)	28 (90.3)		3 (9.7)	9 (75)	1 (8.3)	2 (16.7)	1 (50)		1 (50)			6 (100)	11 (28.2)	10 (25.6)	18 (46.2)
Penicillin			2 (100)	18 (35.3)	3 (5.9)	30 (58.8)	26 (23.2)	13 (11.6)	73 (65.2)									
Ciprofloxacin	66 (40.5)	14 (8.6)	83 (50.9)	22 (52.4)	4 (9.5)	16 (38.1)	46 (66.7)	7 (10.1)	16 (23.2)	1 (33.3)		2 (66.7)	6 (85.7)		1 (14.3)	27 (64.3)	2 (4.8)	13 (31)
Cotrimoxazole	63 (24.9)	3 (1.2)	187 (73.9)	15 (37.5)	1 (2.5)	24 (60)	8 (44.4)	1 (5.6)	9 (50)		1 (25)	3 (75)	2 (28.6)		5 (71.4)	18 (40.9)	1 (2.3)	25 (56.8)
Ceftazidime	9 (37.5)	50 (20.8)	10 (41.7)				1 (50)		1 (50)				2 (100)			4 (22.2)		14 (77.8)
Ofloxacin	34 (37.4)	2 (2.2)	55 (60.4)			1 (100)			2 (100)	2 (50)		2 (50)	4 (66.7)		2 (33.3)	15 (55.6)	2 (7.4)	10 (37)
Cefixime	72 (35.3)	10 (4.9)	122 (58.9)		1 (25)	3 (75)	2 (16.7)		10 (83.3)	2 (50)		2 (50)	1 (11.1)		8 (88.9)	9 (31)	1 (3.4)	19 (65.5)
Cephalothin	17 (28.8)	4 (6.8)	38 (64.4)	1 (50)		1 (50)	11 (55)	3 (15)	6 (30)			2 (100)			4 (100)	2 (28.6)		5 (71.4)
Amikacin	120 (73.2)	27 (16.5)	17 (10.4)	13 (68.4)	3 (15.8)	3 (15.8)	77 (82.8)	5 (5.4)	11 (11.8)				2 (100)			15 (51.7)	4 (13.8)	10 (34.5)
Ceftriaxone	88 (37.6)	14 (6)	132 (56.4)			1 (100)		1 (50)	1 (50)	1 (100)					2 (100)	12 (48)	5 (20)	8 (32)
Gentamycin	220 (69.2)	30 (9.4)	68 (21.4)	23 (51.1)	8 (17.8)	14 (31.1)	126 (76.4)	8 (4.8)	31 (18.8)		1 (50)	1 (50)	4 (80)		1 (20)	31 (57.4)	5 (9.3)	18 (33.3)
Cefalexin	19 (21.6)	6 (6.8)	63 (71.6)	4 (66.7)	1 (16.7)	1 (16.7)	26 (55.3)	7 (14.9)	14 (29.8)	1 (33.3)	1 (33.3)	1 (33.3)			6 (100)	7 (25)		21 (75)
Imipenem	13 (48.1)	5 (18.5)	9 (33.3)				2 (100)						2 (100)			4 (44.4)	4 (44.4)	1 (11.1)
Cefotaxime	14 (26.9)	5 (9.6)	33 (63.5)	7 (63.6)	1 (9.1)	3 (27.3)	33 (43.4)	10 (13.2)	33 (43.4)						2 (100)	4 (17.4)	2 (8.7)	17 (73.9)
Cefazolin	3 (9.4)	1 (3.1)	28 (87.5)	5 (45.5)	3 (27.3)	3 (27.3)	19 (45.2)	8 (19)	15 (35.7)						1 (100)	1 (7.1)		13 (92.9)
Ampicillin	8 (14)	12 (21.1)	37 (64.9)	2 (20)	3 (30)	5 (50)	13 (40.6)	6 (18.8)	13 (40.6)						2 (100)	1 (8.3)		11 (91.7)
Co-Amoxiclav				3 (37.5)		5 (62.5)	2 (40)	1 (20)	2 (40)									
Rifampin				10 (83.3)		2 (16.7)	7 (100)											1 (100)
Tetracycline			1 (100)	12 (52.2)	4 (17.4)	7 (30.4)	13 (32.5)	3 (7.5)	24 (60)									
Azithromycin	1 (100)					1 (100)		1 (5.6)	17 (94.4)									
Vancomycin	2 (33.3)	1 (16.7)	3 (50)	4 (17.4)	6 (26.1)	13 (56.5)	39 (32.5)	31 (25.8)	50 (41.7)									
Erythromycin			1 (100)	4 (25)	3 (18.8)	9 (56.3)	9 (27.3)	3 (9.1)	21 (63.6)									
Clindamycin			2 (100)	6 (46.2)	1 (7.7)	6 (46.2)	13 (28.9)	8 (17.8)	24 (53.3)									1 (100)
Nalidixic Acid	33 (21.2)	20 (12.8)	103 (66)									1 (100)			1 (100)	5 (27.8)	8 (44.4)	5 (27.8)
Ceftizoxime	3 (33.3)	2 (22.2)	4 (44.4)	3 (100)			3 (37.5)		5 (62.5)									1 (100)
Cefoxitin	2 (28.6)		5 (71.4)	14 (34.1)	3 (7.3)	24 (58.5)	9 (69.2)		4 (30.8)						2 (100)			
Amoxicillin	17 (12.1)	6 (4.3)	117 (83.6)	4 (23.5)	5 (29.4)	8 (47.1)	11 (45.8)	2 (8.3)	11 (45.8)		1 (100)				1 (100)			20 (100)
Cefepime						1 (100)				1 (50)		1 (50)	4 (100)			3 (60)		2 (40)
Doxycycline				4 (66.7)		2 (33.3)	1 (100)											
Oxacillin	12 (50)	5 (20.8)	7 (29.2)			2 (100)			1 (100)									

**Table 5:** Antibiotic resistance patterns based on pathogens (cont.)

Antibiotic; n (%)	*Streptococcus*			*Enterobacter*			Alkalogenic bacteria			*Citrobacter*			*Flavobacteria*			
	Sensitive	Intermediate	Resistant	Sensitive	Intermediate	Resistant	Sensitive	Intermediate	Resistant	Sensitive	Intermediate	Resistant	Sensitive	Intermediate	Resistant	
Norfloxacin		1 (100)		6 (85.7)		1 (14.3)										
Nitrofurantoin	1 (100)			1 (12.5)	3 (37.5)	4 (50)					1 (50)	1 (50)				
Penicillin	6 (26.1)	2 (8.7)	15 (65.2)													
Ciprofloxacin	5 (55.6)	1 (11.1)	3 (33.3)	3 (50)	1 (16.7)	2 (33.3)			1 (100)				1 (100)			
Cotrimoxazole	1 (14.3)	1 (14.3)	5 (71.4)	8 (80)		2 (20)			1 (100)			1 (100)				
Ceftazidime		1 (50)	1 (50)						1 (100)	1 (100)					1 (100)	
Ofloxacin				1 (50)		1 (50)						1 (100)				
Cefixime			2 (100)	3 (42.9)	1 (14.3)	3 (42.9)						1 (100)				
Cephalothin			3 (100)			1 (100)						1 (100)				
Amikacin	6 (42.9)	1 (7.1)	7 (50)	2 (50)	2 (50)				1 (100)	1 (100)					1 (100)	
Ceftriaxone				6 (54.5)		5 (45.5)			1 (100)	1 (100)						
Gentamycin	9 (34.6)	4 (15.4)	13 (50)	9 (75)	1 (8.3)	2 (16.7)				2 (50)		2 (50)			1 (100)	
Cefalexin			4 (100)			2 (100)						1 (100)				
Imipenem			1 (100)							3 (100)				1 (100)		
Cefotaxime	7 (70)	1 (10)	2 (20)						1 (100)	2 (66.7)		1 (33.3)			1 (100)	
Cefazoline	1 (33.3)		2 (66.7)						1 (100)			1 (100)				
Ampicillin	2 (66.7)		1 (33.3)						1 (100)	1 (50)		1 (50)				
Co-Amoxiclav																
Rifampin																
Tetracycline		1 (100)														
Azithromycin			3 (100)													
Vancomycin	7 (35)	4 (20)	9 (45)													
Erythromycin	1 (7.7)	1 (7.7)	11 (84.6)													
Clindamycin	3 (50)	1 (16.7)	2 (33.3)													
Nalidixic Acid				3 (42.9)	1 (14.3)	3 (42.9)										
Ceftizoxime	2 (33.3)		4 (66.7)													
Cefoxitin	1 (100)															
Amoxicillin						9 (100)										
Cefepime									1 (100)							
Doxycycline	2 (100)															
Oxacillin			2 (100)													

## Discussion

Iran is one of the countries with high consumption of antimicrobials which could be due to the uncontrolled, over-the-counter sale of medicines, including antimicrobials [11, 12]. Researchers warned of antimicrobial overuse and overprescribing and the resulting AMR as a major hurdle for Iran’s health system half a century ago [13]. This is still a source of concern, as the National Committee for Rational Use of Drugs (NCRUD) reported in 2015 that more than half of patients received antibiotics which are considered irrational behavior by NCRUD [11]. Accordingly, this cross-sectional study has shown high levels of resistance to recommended antibiotics in the ambulatory setting in four different groups of bacteria, including the *Enterobacteriaceae* family, *Pseudomonas aeruginosa, Coagulase-negative staphylococci, and Coagulase positive staphylococci*.

## The Enterobacteriaceae family

Gram-negative pathogens cause significant morbidity [7]. The *Enterobacteriaceae* family and *Pseudomonas aeruginosa* are motile gram-negative rod-shaped pathogens studied in this article [2]. The *Enterobacteriaceae* family, including *E. coli*, *Klebsiella spp., and Enterobacter spp.*, is the leading cause of urinary tract infections (UTIs), bloodstream infections, hospital infections, and healthcare-associated pneumonia [14, 15]. Most of the Enterobacteriaceae samples used in this study are isolated from urine, indicating the role of this group of bacteria in causing UTIs. There were also fewer samples from blood and throat containing *E. coli* and *Klebsiella*.

In case of treatment, Nitrofurantoin, fluoroquinolones, and cephalosporins are usually considered appropriate choices for empirical therapy of bacteria belonging to the *Enterobacteriaceae family*, although third and earlier generations of cephalosporines are no longer a desirable option [16].

This trial shows a low rate of resistance (8.5%) against Nitrofurantoin in *E. coli*, but it is not recommended in the treatment of *Klebsiella* or *Enterobacter* species considering the high rate of resistance of around 50% to this antibiotic. Norfloxacin, Ciprofloxacin, and Ofloxacin were investigated from fluoroquinolones group of antibiotics. They are still pretty much effective against this family but Ciprofloxacin and Ofloxacin are not recommended for *E. coli* infection.

Resistance to third-generation cephalosporins in Enterobacteriaceae is caused by the production of Beta-lactamases [17]. All three subgroups of Enterobacteriaceae studied are approximately 50% resistant to this generation of cephalosporins. Ceftriaxone had the lowest rate of resistance (32%) in *Klebsiella*.

Resistance to Ampicillin, Amoxicillin, and early generation cephalosporins is caused by class A Beta-lactamase enzymes [18]. Based on results of this study, these antibiotics are not recommended for empirical antibiotic therapy of UTI either considering high resistance, around 80%.

In summary, based on our study Aminopenicillin family and earlier than the third generation of cephalosporin are not effective against this family of bacteria, and the 3rd generation of cephalosporin is at risk of getting resistant.

### Pseudomonas aeruginosa

*Pseudomonas aeruginosa* is a gram-negative aerobic bacterium found in the normal intestinal flora and a potent pathogen that causes infection in immunocompromised patients [19]. This pathogen can survive on dry hospital surfaces. It is one of the most common nosocomial pathogens that can cause ventilator-associated pneumonia and bloodstream infections [20].

Carbapenem, a class of antibiotics commonly used when bacteria are resistant to cephalosporins and fluoroquinolones, are B-lactam antibiotics that inhibit the synthesis of bacterial peptidoglycan cell walls can be effective in the treatment of patients with *Pseudomonas aeruginosa (P. aeruginosa)* [21]. In our study, only two samples of *P. aeruginosa* were tested regarding their sensitivity to Imipenem, in which both were sensitive. Other studies have also reported a 94.15% sensitivity to Imipenem has been reported [22]. However, due to our small sample size, further larger populational studies are required to draw a clear conclusion regarding the sensitivity and resistance of B-lactam antibiotics among *P. aeruginosa*.

Other studies show that Cephalosporins of the third and fourth generations, such as Ceftazidime and Cefepime, are the most effective Beta-lactams used in the treatment of *P. aeruginosa* [21]. A study by Fazeli et al. reported a 24.6% sensitivity to Ceftazidime has been reported [23]. The samples evaluating sensitivity of Ceftazidime, Cefotaxime, and Ceftriaxone in our study were limited and provide efficient data to estimate the AMR among *P. aeruginosa*. However, we cannot deny the uprising of resistance of the mentioned antibiotics in this microorganism.

Resistance to aminoglycosides in *P. aeruginosa* is mediated by transferable aminoglycoside modifying enzymes (AMEs) [21, 24]. However, in Iran and our study, *P. aeruginosa* is still sensitive to Amikacin, and there is only 20% resistance to Gentamycin.

### Coagulase-negative staphylococci (CoNS)

*Coagulase-negative staphylococci (CoNS)* are a large group of gram-positive cocci distinguished by the absence of the coagulase virulence factor [25]. *CoNS* are common skin and mucosa microflora that coexists with *S. aureus* and various other bacteria in the human nostrils [26]. Many *CoNS* infections are caused by foreign bodies, which promote biofilm colonization and contribute to *CoNS* pathogenic potential [27]. They are usually categorized as contaminants instead of infectious agents [28].

Macrolides are one of the most commonly prescribed antibiotics against this group in ambulatory settings [29]. We discovered a concerning high rate of Azithromycin (94.4%) and Erythromycin (63.6%) resistance. In a previous study reported by Asante et al. 74.2% of all isolates were resistant to Azithromycin and Erythromycin [30].

The most common species of *Coagulase positive staphylococci* is *Staphylococcus aureus (S. aureus).* It is a major pathogen in hospital and community-acquired acquired infections, and it can cause a variety of infectious diseases, including mild skin and soft tissue infections, infective endocarditis, bacteremia, and osteomyelitis [31]. *S. aureus* was well-managed up until 1950, when penicillin resistance developed. Then methicillin was introduced to clinical practice, which was effective in treating penicillin-resistant *S. aureus* infections [32, 33]. Vancomycin has long been considered as the last line of treatment against gram-positive cocci infection [34]. However, *Staphylococcus aureus* resistance to Vancomycin is rising on a daily basis, causing major issues in the medical community [35]. The 56.5% resistance of positive staph coagulase to Vancomycin is accompanied with clinical significance, based on the challenges in the management and mortalities associated with vancomycin resistant infections [36].

### Age-related rate of antimicrobial resistance

The elderly are a notably important population in terms of antibiotic overuse, using roughly 50% more antibiotics than younger adults [37, 38]. This overuse can be due to a combination of factors, including physiological changes leading to recurrent infections and frequent exposure to multidrug resistance bacteria in long-term care facilities [39–43]. The higher number of comorbidities among older patients causes more hospitalizations, a setting where they get exposed to multidrug-resistant bacteria [44, 45]. as a result of repeated exposure to multidrug resistant bacteria and higher risk of infection, AMR rates are approximately 2 to 3-folds higher in older patients comparing to younger patients [43]. In our research, high AMR is shown in patients older than 60-year-old. This case can be due to arbitrary use of antibiotics, aging and health problems in these people. Also, empirical antibiotic administration and overuse of antibiotic in patients without any clinical indications is a growing concern in our country, which overtime can increase AMR [46, 47]. The growing resistance to antibacterial is obvious comparing the age groups of 7 to 18 and the 19 to 60.

### Gender-related rate of antimicrobial resistance

Although women are approximately 27% more likely than men to receive antibiotic prescriptions, AMR is higher among men. This can be due to biological differences, differences in prescribed antibiotics, and more likelihood of men contracting hospital-acquired infections due to higher hospitalization rates, especially in older age groups. Poorer compliance of men for using medication or hand hygiene recommendations can also contribute to this difference [48–52]. Microbiological resistance pattern among male and female patients has been compared in Fig. 2. Our study also shows lower AMR among females, although they make up the majority of individuals receiving antibiotics in this study (54.5%).

### Ward-related rate of antimicrobial resistance

Our study demonstrated that resistance with Cephalexin, Cefotaxime, Cefazolin, and also Amoxicillin were the most frequent in the ICU, Cephalexin, Penicillin in infectious, and also Cephalexin, Cefixime in surgery. Other studies have also supported the increase of resistance of cephalosporins and fluroquinolones in ICUs, which are mainly due to use of invasive procedures and overall hospital-acquired infections rate [53]. Gong et al. stated that infection profiles in ICU and wards differ, and multidrug resistance in ICU is more severe than in wards. As a result, various infection-control measures should be prioritized in various populations [54].

### Limitations

Among the limitations was the retrospective and cross-sectional nature of our study, which limits us in evaluating the possible causative relationship between the patients features and conditions with their microorganism resistance patterns. Also, our study lacks molecular analysis to identify the source of infection was required. Another limitation is the small sample size in a number of evaluated microorganisms, which limits the capability of providing a generalizable estimate of the sensitivity percentage among our study population. Further multicentral studies are required along with the evaluation of applied hospital infection control policies to increase the understanding of the trend of microorganism resistance patterns and possible effective mechanism in the control and management of this.

## Conclusion

In this study conducted in Yasuj Hospitals, Southern west Iran, we isolated different microorganisms from different wards and surveyed their rate of antibiotic resistance. Considering widespread empirical antibiotic therapy in Iran, the rate of increasing resistance to common antibiotics prescribed for ambulatory and hospitalized patients is concerning. Aminopenicillin family and earlier than the third generation of cephalosporin are not recommended for the treatment of the Enterobacteriaceae family. *P. aeruginosa* is sensitive to Amikacin, but it is developing multidrug resistance globally. In Coagulase-negative staphylococci, Macrolides are usually prescribed, but high rates of resistance against this group of antibiotics have developed. Considering *S. aureus*, Vancomycin resistance is higher than 50%. Since this medication is the last resort in the treatment of Methicillin-resistant *S. aureus* this level of resistance is alarming. We should also take into account AMR threats against elderly population since it leads to more severe disease and prolonged hospitalization in this group. Also, reasons of the high rate of AMR in men should be investigated more accurately. We recommend for demonstrating a plan to control overuse and over prescription of antimicrobials by health policy making organization.

## Data Availability

All data regarding this study has been reported in the manuscript. Please contact the corresponding author if you are interested in any further information.

## Abbreviations

AME: Aminoglycoside Modifying Enzyme
AMR: Antimicrobial Resistance
CLSI: Clinical and Laboratory Standard Institute
Cons: Coagulase-Negative Staphylococci
CSF: Cerebrospinal Fluid
E.Coli: Escherichia Coli
ETT: Endotracheal Tube
ICU: Intensive Care Unit
NCRUD: National Committee for Rational Use of Drugs
P. Aeruginosa: Pseudomonas Aeruginosa
SD: Standard Deviation
S. Aureus: Staphylococcus Aureus
UTI: Urinary Tract Infections

## References

[1] Misganaw A, Haregu TN, Deribe K, Tessema GA, Deribew A, Melaku YA, Amare AT, Abera SF, Gedefaw M, Dessalegn M (2017). National mortality burden due to communicable, non-communicable, and other diseases in Ethiopia, 1990–2015: findings from the global burden of Disease Study 2015. Popul Health Metr.

[2] Retamar P, Portillo MM, Lopez-Prieto MD, Rodriguez-Lopez F, de Cueto M, Garcia MV, Gomez MJ, Del Arco A, Munoz A, Sanchez-Porto A (2012). Impact of inadequate empirical therapy on the mortality of patients with bloodstream infections: a propensity score-based analysis. Antimicrob Agents Chemother.

[3] Ahmadishooli A, Davoodian P, Shoja S, Ahmadishooli B, Dadvand H, Hamadiyan H, Shahriarirad R (2020). Frequency and Antimicrobial susceptibility patterns of Diabetic Foot infection of patients from Bandar Abbas District, Southern Iran. J Pathog.

[4] Vazin A, Shahriarirad R, Azadeh N, Parandavar N, Kazemi K, Shafiekhani M. Incidence, clinicomicrobiological characteristics, risk factors, and TreatmentOutcomes of bacterial infections following liver transplantation in Pediatrics: a retrospective cohort study. Archives of Pediatric Infectious Diseases 2022 (In Press).

[5] Where Resistance Spreads. : Across the World | CDC. [https://www.cdc.gov/drugresistance/across-the-world.html].

[6] Laxminarayan R, Duse A, Wattal C, Zaidi AK, Wertheim HF, Sumpradit N, Vlieghe E, Hara GL, Gould IM, Goossens H (2013). Antibiotic resistance-the need for global solutions. Lancet Infect Dis.

[7] Breijyeh Z, Jubeh B, Karaman R (2020). Resistance of Gram-Negative Bacteria to current Antibacterial Agents and Approaches to resolve it. Molecules.

[8] Tille PM. Bailey and Scott’s Diagnostic Microbiology. 2017.

[9] Hudzicki J. Kirby-Bauer disk diffusion susceptibility test protocol.

[10] Wayne P. Clinical and laboratory standards institute. Performance standards for antimicrobial susceptibility testing. 2011. 31339681

[11] Abbasian H, Hajimolaali M, Yektadoost A, Zartab S (2019). Antibiotic utilization in Iran 2000–2016: Pattern Analysis and Benchmarking with Organization for Economic Co-operation and Development Countries. J Res Pharm Pract.

[12] Hashemi S, Nasrollah A, Rajabi M (2013). Irrational antibiotic prescribing: a local issue or global concern?. EXCLI J.

[13] Amidi S, Solter S, Rashidian B, Zokaian A-R, Razmjoian F. Antibiotic use and abuse among physicians in private practice in Shiraz, Iran. Med Care 1975:341–5. 10.1097/00005650-197504000-00006 1121196

[14] Brenner DJ, Farmer JJ. <i> Enterobacteriaceae</i>. Bergey’s Manual of Systematics of Archaea and Bacteria. Wiley; 2015: 1–24.

[15] Janda JM, Lopez DL. The Family Enterobacteriaceae. Practical handbook of Microbiology. CRC Press; 2021: 353–62.

[16] Brusch J, Bronze M (2020). Urinary tarct infection (UTI) and cystitis (bladder infection) in females. Child Urin Tract Infect.

[17] Iredell J, Brown J, Tagg K (2016). Antibiotic resistance in Enterobacteriaceae: mechanisms and clinical implications. BMJ.

[18] Kapmaz M, Erdem F, Abulaila A, Yeniaras E, Oncul O, Aktas Z (2016). First detection of NDM-1 with CTX-M-9, TEM, SHV and rmtC in Escherichia coli ST471 carrying IncI2, A/C and Y plasmids from clinical isolates in Turkey. J Glob Antimicrob Resist.

[19] Wu W, Jin Y, Bai F, Jin S. Pseudomonas aeruginosa. Molecular Medical Microbiology. Elsevier; 2015: 753–67.

[20] Lyczak JB, Cannon CL, Pier GB (2000). Establishment of Pseudomonas aeruginosa infection: lessons from a versatile opportunist. Microbes Infect.

[21] van Duin D, Doi Y (2017). The global epidemiology of carbapenemase-producing Enterobacteriaceae. Virulence.

[22] Osundiya O, Oladele R, Oduyebo O (2013). Multiple antibiotic resistance (MAR) indices of Pseudomonas and Klebsiella species isolates in Lagos University Teaching Hospital. Afr J Clin Experimental Microbiol.

[23] Fazeli H, Solgi H, Havaei SA, Shokri D, Norouzi Barogh M, Zamani FZ (2014). Carbapenem and fluoroquinolone resistance in multidrug resistant pseudomonas aeruginosa isolates from al-zahra hospital, isfahan, iran. J Med Microbiol Infect Dis.

[24] Driscoll JA, Brody SL, Kollef MH (2007). The epidemiology, pathogenesis and treatment of Pseudomonas aeruginosa infections. Drugs.

[25] Becker K, Both A, Weisselberg S, Heilmann C, Rohde H (2020). Emergence of coagulase-negative staphylococci. Expert Rev Anti Infect Ther.

[26] Kock R, Werner P, Friedrich AW, Fegeler C, Becker K (2016). Prevalence of Multiresistant Microorganisms Study G, Prevalence of Multiresistant Microorganisms PMMSG: persistence of nasal colonization with human pathogenic bacteria and associated antimicrobial resistance in the german general population. New Microbes New Infect.

[27] Hussain M, Kohler C, Becker K (2020). Role of SrtA in pathogenicity of Staphylococcus lugdunensis. Microorganisms.

[28] Michels R, Last K, Becker SL, Papan C (2021). Update on coagulase-negative staphylococci-what the Clinician should know. Microorganisms.

[29] Marincola G, Liong O, Schoen C, Abouelfetouh A, Hamdy A, Wencker FDR, Marciniak T, Becker K, Kock R, Ziebuhr W (2021). Antimicrobial Resistance Profiles of Coagulase-Negative Staphylococci in Community-Based healthy individuals in Germany. Front Public Health.

[30] Asante J, Hetsa BA, Amoako DG, Abia ALK, Bester LA, Essack SY (2021). Multidrug-resistant coagulase-negative Staphylococci isolated from Bloodstream in the uMgungundlovu District of KwaZulu-Natal Province in South Africa: emerging pathogens. Antibiot (Basel).

[31] Humphreys H (2012). Staphylococcus aureus: the enduring pathogen in surgery. Surgeon.

[32] Jokinen E, Laine J, Huttunen R, Rahikka P, Huhtala H, Vuento R, Vuopio J, Syrjanen J (2017). Comparison of outcome and clinical characteristics of bacteremia caused by methicillin-resistant, penicillin-resistant and penicillin-susceptible Staphylococcus aureus strains. Infect Dis (Lond).

[33] Pichereau S, Rose WE (2010). Invasive community-associated MRSA infections: epidemiology and antimicrobial management. Expert Opin Pharmacother.

[34] Micek ST (2007). Alternatives to vancomycin for the treatment of methicillin-resistant Staphylococcus aureus infections. Clin Infect Dis.

[35] Haseeb A, Ajit Singh V, Teh CSJ, Loke MF (2019). Addition of ceftaroline fosamil or vancomycin to PMMA: an in vitro comparison of biomechanical properties and anti-MRSA efficacy. J Orthop Surg (Hong Kong).

[36] DiazGranados CA, Zimmer SM, Klein M, Jernigan JA (2005). Comparison of mortality associated with vancomycin-resistant and vancomycin-susceptible enterococcal bloodstream infections: a meta-analysis. Clin Infect Dis.

[37] Arizpe A, Reveles KR, Aitken SL (2016). Regional variation in antibiotic prescribing among medicare part D enrollees, 2013. BMC Infect Dis.

[38] Hicks LA, Bartoces MG, Roberts RM, Suda KJ, Hunkler RJ, Taylor TH, Schrag SJ (2015). US outpatient antibiotic prescribing variation according to geography, patient population, and provider specialty in 2011. Clin Infect Dis.

[39] Beckett CL, Harbarth S, Huttner B (2015). Special considerations of antibiotic prescription in the geriatric population. Clin Microbiol Infect.

[40] El Chakhtoura NG, Bonomo RA, Jump RLP (2017). Influence of aging and environment on presentation of infection in older adults. Infect Dis Clin North Am.

[41] Esme M, Topeli A, Yavuz BB, Akova M (2019). Infections in the Elderly critically-ill patients. Front Med (Lausanne).

[42] Ittisanyakorn M, Ruchichanantakul S, Vanichkulbodee A, Sri-On J (2019). Prevalence and factors associated with one-year mortality of infectious diseases among elderly emergency department patients in a middle-income country. BMC Infect Dis.

[43] O’Fallon E, Kandel R, Schreiber R, D’Agata EM (2010). Acquisition of multidrug-resistant gram-negative bacteria: incidence and risk factors within a long-term care population. Infect Control Hosp Epidemiol.

[44] Pop-Vicas AE, D’Agata EM (2005). The rising influx of multidrug-resistant gram-negative bacilli into a tertiary care hospital. Clin Infect Dis.

[45] Tumbarello M, Sali M, Trecarichi EM, Leone F, Rossi M, Fiori B, De Pascale G, D’Inzeo T, Sanguinetti M, Fadda G (2008). Bloodstream infections caused by extended-spectrum-beta-lactamase- producing Escherichia coli: risk factors for inadequate initial antimicrobial therapy. Antimicrob Agents Chemother.

[46] Nimer NA (2022). Nosocomial infection and antibiotic-resistant threat in the Middle East. Infect Drug Resist.

[47] Hadi U, Kolopaking EP, Gardjito W, Gyssens IC, Van den Broek P (2006). Antimicrobial resistance and antibiotic use in low-income and developing countries. Folia Med Indonesiana.

[48] Humphreys H, Fitzpatick F, Harvey BJ (2015). Gender differences in rates of carriage and bloodstream infection caused by methicillin-resistant Staphylococcus aureus: are they real, do they matter and why?. Clin Infect Dis.

[49] Neubeiser A, Bonsignore M, Tafelski S, Alefelder C, Schwegmann K, Ruden H, Geffers C, Nachtigall I (2020). Mortality attributable to hospital acquired infections with multidrug-resistant bacteria in a large group of german hospitals. J Infect Public Health.

[50] Schroder W, Sommer H, Gladstone BP, Foschi F, Hellman J, Evengard B, Tacconelli E (2016). Gender differences in antibiotic prescribing in the community: a systematic review and meta-analysis. J Antimicrob Chemother.

[51] van Lunzen J, Altfeld M (2014). Sex differences in infectious diseases-common but neglected. J Infect Dis.

[52] Vincent JL, Sakr Y, Singer M, Martin-Loeches I, Machado FR, Marshall JC, Finfer S, Pelosi P, Brazzi L, Aditianingsih D (2020). Prevalence and outcomes of infection among patients in Intensive Care Units in 2017. JAMA.

[53] Jacoby TS, Kuchenbecker RS, Dos Santos RP, Magedanz L, Guzatto P, Moreira LB (2010). Impact of hospital-wide infection rate, invasive procedures use and antimicrobial consumption on bacterial resistance inside an intensive care unit. J Hosp Infect.

[54] Gong Y, Peng Y, Luo X, Zhang C, Shi Y, Zhang Y, Deng J, Peng Y, Luo G, Li H (2021). Different infection profiles and antimicrobial resistance patterns between burn ICU and common wards. Front Cell Infect Microbiol.

